# Engineered Approaches to Facile Identification of Tiny Microplastics in Polymeric and Ceramic Membrane Filtrations for Wastewater Treatment

**DOI:** 10.3390/membranes12060565

**Published:** 2022-05-28

**Authors:** Heejin Kook, Chanhyuk Park

**Affiliations:** Department of Environmental Science and Engineering, Ewha Womans University, Seoul 03760, Korea; 202eng13@ewhain.net

**Keywords:** ceramic membrane, tiny microplastics, particle size distribution analysis, polymeric membrane, wastewater

## Abstract

Wastewater treatment plants (WWTPs) contribute to the release of significant quantities of microplastics into the aquatic environment. The facile identification of microplastics and an understanding of their occurrence and transport through WWTPs are essential for improving microplastic retention. Potential microplastic treatment technologies for both polymeric and ceramic membrane filtrations were systematically investigated to inform decisions on the optimal choice of membrane for effective microplastic retention. A blocking filtration model, based on a simple linear regression fitting, was used in experiments on the filtration of microplastic suspensions to determine the relative importance of individual fouling mechanisms. Unlike the commonly applied spectroscopic techniques, the facile identification approaches, that are closely related to the amounts of particles within wastewater samples, attempted to identify tiny microplastics (<1.0 µm) by comparing them against silica particles for reference. A larger decline in the normalized permeate flux was observed for 0.1 μm polystyrene microplastics, while standard pore blocking appeared to be the dominant fouling mechanism for all membranes. More microplastics based on turbidity and total solids were removed using the ceramic membrane than the other polymeric membranes. However, fewer microplastics, based on the particle size distribution analysis, were removed using the ceramic membrane as the pore size measurements gave a relatively large pore size for the ceramic membrane, compared with other polymeric membranes; even though a nominal pore size of 0.1 μm for all membranes were provided by the suppliers. The contribution of microplastic-containing synthetic wastewaters to overall flux decline was significantly greater than those of identical microplastic suspensions because of the aggregation of larger microplastics with dissolved organic matter in synthetic wastewater, leading to the formation of a cake layer on the membrane surface. Despite the challenges associated with the facile identification approaches, our findings provided deeper insights and understanding of how microplastics behave in membrane filtration, which could enable the application of potential microplastic treatment technologies.

## 1. Introduction

Microplastics are generally defined as small plastic pieces, less than 5 mm in length, that can be harmful to aquatic life [1,2]. Primary microplastics are used in many personal care and cosmetic products, while secondary microplastics can be formed from a variety of sources, including larger plastic debris that degrades into progressively smaller pieces. Microbeads contained within facial cleansers and toothpaste, along with the thousands of microplastic fibers dislodged during washing, are often directly discharged into wastewater [3,4]. However, microplastics entering wastewater treatment plants (WWTPs) can be partially treated before being released into the aquatic environment, depending on the treatment processes employed [5,6,7]. The reported types and concentrations of microplastics in influent wastewater samples varied greatly in different WWTPs. The most common types of microplastics detected at WWTPs are polyester (PES, 28–89%), polyethylene (PE, 4–51%), polyethylene terephthalate (PET, 4–35%) and polyamides (PA, 3–30%) along with other polymers, such as acrylate, polypropylene (PP), and polystyrene (PS) (5–27%) [8]. These microplastics can be treated via a series of wastewater treatment processes typically composed of primary clarifiers, biological treatments, and final sedimentation. At present, few existing studies on microplastic removal in WWTPs compare removal efficiency during preliminary, primary, secondary, and tertiary treatments [5,9]. Existing studies have shown that a significant proportion of microplastics are removed by preliminary and primary treatments (pre-treatment) with the removal efficiency dependent on properties, such as the size distribution, shape, and density of the microplastics [6,10,11]. Secondary treatments can further decrease microplastic concentrations in wastewater and can effectively remove more fragment particles than fibers, while tertiary treatments may provide substantial additional polishing of microplastics before release, depending on the treatment process [6,11,12]. A significant proportion of microplastics (>20 µm) are effectively removed in advanced final-stage wastewater treatment. Membrane-related technologies demonstrate the highest removal efficiency (99.9%), followed by rapid sand filters (RSFs) and dissolved air flotation (DAF), with a removal efficiency of 97% and 95%, respectively [9]. When using membranes with a nominal pore size of 0.4 µm, microplastics concentrations decreased from 6.9 ± 1.0 to 0.005 ± 0.004 particles/L [5]. Recent studies have also shown that the relative abundance of microplastics decreased when membrane bioreactor (MBR) processes are used in wastewater treatment [13,14,15]. Emerging applications involving alternative membrane materials, such as ceramic membranes, have been increasing in recent years, as they offer greater permeate flux and lower fouling propensity to a variety of wastewater treatments through their membrane-based processes [16,17,18,19].

Microplastic analysis can be classified into physical and chemical characterizations [5]. Chemical characterizations are mainly used to determine the composition of microplastics and can increase the accuracy of microplastic identification and further explore their composition. Current chemical analysis methods include destructive techniques, such as gas chromatography coupled to mass spectrometry (GC-MS), which includes pyrolysis-GC-MS and thermal extraction desorption-GC-MS [20,21,22,23,24], and non-destructive spectroscopic techniques, such as Fourier transform infrared (FTIR) [25,26,27] and Raman spectroscopy [28,29]. Among these techniques, spectroscopic approaches are most commonly used to identify microplastics contained in environmental samples, although equipment limitations make it difficult to detect tiny microplastics (<1 µm) [30,31]. FTIR is the most frequently reported method used in the analysis of microplastics found in WWTPs. However, traditional FTIR analysis is very labor-intensive as the microplastics first need to be identified under light microscope, and then the spectrum of each particle needs to be individually analyzed. The recent development of focal plane array (FPA)- based micro-FTIR imaging may be more effective in evaluating the spectra of individual particles in a wastewater sample, resulting in high-throughput analysis of the total microplastic contents [26,32]. However, the micro-FTIR technique is still limited to specific diffraction ranges (e.g., 10 µm at 1000 cm^−1^) and samples of 10–20 µm in size can rarely be analyzed [2]. Compared with FTIR, Raman techniques can give a better spatial resolution (down to 1 µm) [33,34]. However, care must be taken when purifying the samples in order to avoid accidental sample modification prior to analysis [35]. Unlike chemical characterization methods, physical characterization mainly refers to characterizing the size distribution of microplastics, as well as assessing other physical parameters [36]. The technique can be used to rapidly measure the morphology of small-sized microplastics using relatively inexpensive equipment. Additionally, as it requires no pre-treatment, it is less labor-intensive. Although the method cannot determine specific polymer types, and cannot eliminate potential errors, the facile and indirect quantification approaches for identifying the microplastics, such as turbidity, mass, and size distribution may be able to highlight the importance of determining microplastic transport through WWTPs. Therefore, several water quality parameters that are used to assess the quality of wastewater discharged into the environment were suggested for use in characterizing the properties of microplastic particles in wastewater samples [37,38,39].

The present study addresses the potential of several engineered facile and indirect identification approaches by examining the removal efficiency and behavior of microplastics during wastewater treatment using membrane processes. Two tiny, differently sized PS and PE (0.1 and 1.0 µm) microplastics that cannot be measured by other spectroscopic techniques were chosen as the target microplastics. based on their high levels of prevalence in WWTPs, and their transport mechanisms were compared with standard silica particles. By applying several facile microplastic identification methods, the study aimed to fulfil the following specific objectives: (i) to investigate the effects of different types of particles (silica particle and microplastic), (ii) the effects of different sizes of microplastics, (iii) the effects of different types of microplastics (PS and PE), and (iv) the effects of synthetic wastewater samples on filtration and treatment behaviors. The results are expected to offer valuable insights into how we operate a membrane treatment system to improve the retention of microplastics, by better understanding the transport of these relatively tiny microplastics.

## 2. Materials and Methods

### 2.1. Silica Particle and Microplastic

Non-functionalized silica microsphere particle with natural hydroxyl or silanol groups was purchased from EPRUI Biotech Co., Ltd. (Shanghai, China) as a reference particle. The silica microspheres of 0.1 μm nominal diameters were supplied in 10% solids (*w*/*w*) aqueous suspensions. Monodispersed PS microplastics of two different sizes (0.1 and 1.0 μm) at 2% solids (*w*/*w*) aqueous suspensions (Sigma-Aldrich, St. Louis, MO, USA) were used, since they are a particle size standard and are ideal for characterizing the particle size distribution of the samples. PE microplastics with 1.0 μm (Cospheric LLC, Santa Barbara, CA, USA) in dry powder form were suspended in an aqueous solution with a surfactant (Tween 80, Cospheric LLC, Santa Barbara, CA, USA) of 0.5 mg/L prior to being suspended in deionized (DI) water (Direct-Q^®^ 3 Water Purification System, Millipore Corp., Billerica, MA, USA), since PE microplastics are hydrophobic. The concentrations of silica particles and microplastics were suspended in DI water at 50 mg/L for all experiments. For the wastewater samples, the same concentrations of each PS and PE microplastics were added to the prepared synthetic wastewater samples that were adapted from previous studies to maintain the consistency of wastewater composition [40]. 

### 2.2. Membrane Filtration Experiments

Two polyvinylidene fluoride (PVDF) membranes (Synder, Sterlitech Corp., Kent, WA, USA and SteriLUX^®^, Meissner Filtration Products, Camarillo, CA, USA) and the Anopore inorganic membrane (Anodisc, Whatman^®^ Inc., Maidstone, UK), with a 13.1 cm^2^ effective surface area, were employed in a bench-scale membrane filtration system [41,42]. The Anopore inorganic membrane is composed of a high-purity alumina matrix, which is manufactured electrochemically and is hydrophilic, so as to be compatible with most solvents. All membranes were peripherally bonded to an annular polypropylene ring, except for the 13 mm diameter disc, for ease of handling and were suitable for both vacuum and pressure filtration. Pore sizes and the materials (for the top layer) provided by the membrane suppliers are shown in Table 1. 

The pore sizes given by the membrane suppliers were similar to each other. However, the actual membrane pore sizes were determined by measuring the retention of polyethylene oxides (PEO, Sigma-Aldrich, St. Louis, MO, USA) with several different molecular weights, ranging from 100 kDa to 5000 kDa. The molecular weight cut-off (MWCO) of the membranes refers to the minimal molecular weight of organic solutes (i.e., PEO in this study), where 90% of the solute can be retained [18]. The membranes were filtered with 3 g/L PEO solutions at 1.0 bar for 30 min using a peristaltic pump (GT-150D, Green Tech Co., Ltd., Gumi-si, Korea) and PEO retention was determined by measuring the non-purgeable organic carbon (NPOC) concentration in the feed and the permeate, using a total organic carbon (TOC) analyzer (TOC-LCPH, Shimadzu Corp., Kyoto, Japan) [41,42]. The MWCOs in Daltons were converted to metric size (nm) using the Einstein–Stokes diameter equation for PEO according to the Equation (1) [43].

(1) $$R_{s}\;(\mathrm{nm})=0.01044\times {M_{w}}^{0.587}$$

where *R_s_* is the Stokes radius (nm) and *M_w_* is the MWCO (Da). 

Atomic force microscopy (AFM; HR-AFM, AFM Workshop Corp., Hilton Head Island, SC, USA) was used to measure the surface roughness of the membranes in non-contact mode with a 10 μm × 10 μm scale. The porosity was measured using an AutoPore IV 9500 mercury poremeter (Micromeritics Instrument Corp., Norcross, GA, USA), based on the intrusion of mercury into a porous membrane structure under stringently controlled pressures. For these measurements the membranes were cut into small pieces, approximately 10 mm in length. 

The membrane filtration experiments were performed with identical silica particles and microplastic-containing aqueous solutions. A peristaltic pump provided an accurate constant feed flow during all the experiments at a constant transmembrane pressure (TMP) of 1.0 bar, which was maintained by a pressure gauge in front of the membrane. The permeate samples were collected in a glass beaker that was constantly being weighed, using an automated electronic scale (GX-4000, A&D Co., Ltd., Tokyo, Japan), with the weight of the changing volume indicating flux changes. Throughout the experiment, 2 L of feed was stirred at 100 rpm, using a magnetic stirrer (MSH-20A, DAIHAN Scientific Co., Ltd., Wonju, Korea). All the experiments were carried out in duplicate. Firstly, the samples were filtered with DI water to remove contaminants from the system, then the feed water was replaced with ordinary water for the test. The subsequent filtration experiment lasted approximately 2.5 h and all experiments were performed at room temperature (20.8 ± 0.6 °C).

### 2.3. Analytical Methods

Samples for analysis were collected regularly from the feed and permeate in the bench-scale membrane filtration unit. Several physicochemical parameters related to particle concentration were measured in the aqueous solutions. The feed and permeate concentrations were used to calculate the retention, which represents the amounts of particles retained by the membranes. Turbidity was measured using a portable turbidimeter (2100Q, Hach Company, Loveland, CO, USA). The TS were measured by weighing the amounts of solids present in a known volume of sample in accordance with the Standard Methods 2540 D/E (APHA et al., 1992). The method involved weighing a beaker, filling it with a known volume, evaporating the water in an oven and completely drying the residue, and then weighing the beaker with the residue. The TS concentration was equal to the difference between the weight of the beaker with the residue and the weight of the beaker without it. The size distribution of the particles was measured using a Mastersizer 3000 (Malvern Panalytical Ltd., Malvern, UK).

### 2.4. Fouling Mechanisms

Blocking filtration models describe the four mechanisms of membrane fouling by colloidal particles (Figure S1 in Supplementary Material) as (a) complete pore blocking, (b) standard pore blocking, (c) intermediate pore blocking, and (d) cake filtration [44,45]. Complete pore blocking occurs when a particle reaching the membrane blocks a pore entrance without superimposing over other particles when the particle sizes are similar to the nominal pore size of the membrane. Standard pore blocking occurs when particles are deposited within the pores, resulting in a decrease in the pore volume. Intermediate pore blocking indicates that some particles deposit on other particles, while other particles block membrane pores as represented by complete pore blocking. Cake filtration allows the accumulation of deposited particles on the membrane surface since the membrane pores are already covered by other particles [46]. For membrane filtration carried out in a constant TMP mode with spherically shaped foulants that are completely retained, the equations describing the relationship between the total filtered volume (*V*), and filtration time (*t*), for the individual fouling mechanisms are shown below [44].

(2) $$K_{b}V\;=Q_{0}\left(1-e^{-K_{b}t}\right)\;\left(\mathrm{Complete}\;\mathrm{pore}\;\mathrm{blocking}\right)$$

(3) $$\frac{K_{s}t}{2}=\frac{t}{V}-\frac{1}{Q_{0}}\;(\mathrm{Standard}\;\mathrm{pore}\;\mathrm{blocking})$$

(4) $$K_{i}V=ln\left(1+K_{i}Q_{0}t\right)\;\left(\mathrm{Intermediate}\;\mathrm{pore}\;\mathrm{blocking}\right)$$

(5) $$K_{c}V=\frac{2t}{V}-\frac{2}{Q_{0}}\;\left(\mathrm{Cake}\;\mathrm{filtration}\right)$$

where *Q*_0_ is the initial flow rate and *K* is the constant with the subscript indicating the blocking mechanism. 

An alternative approach to identifying colloidal fouling mechanisms involves applying the blocking filtration models in their integrated forms, which involves a straightforward linear least-square fit and allows for facile identification of individual fouling mechanisms. Clear identification and differentiation between the pore blocking mechanisms has important practical implications, as knowing the fouling mechanism can determine the optimal choice of membrane. In this study, we performed dead-end filtration experiments, using polymeric and ceramic membranes for retaining tiny microplastics, before fitting Equations (2)–(5) to the experimental flux data using linear least-square fitting to identify the relative importance of individual fouling mechanisms.

## 3. Results and Discussion

### 3.1. Effect of Different Types of Particles on the Filtration and Treatment Performance

**Figure 1:**
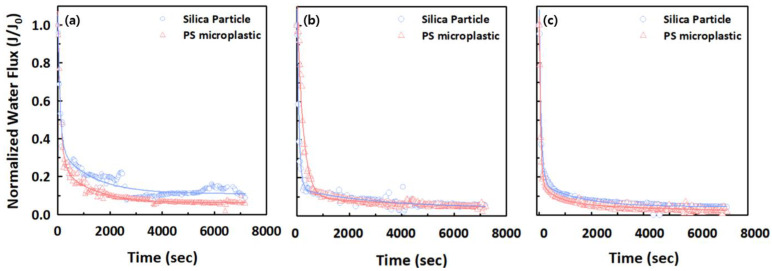
Normalized water flux decline of silica and PS microplastic particles with an average size of 0.1 μm for (**a**) Synder, (**b**) SteriLUX^®^, and (**c**) Anodisc membranes.

**Figure 2:**
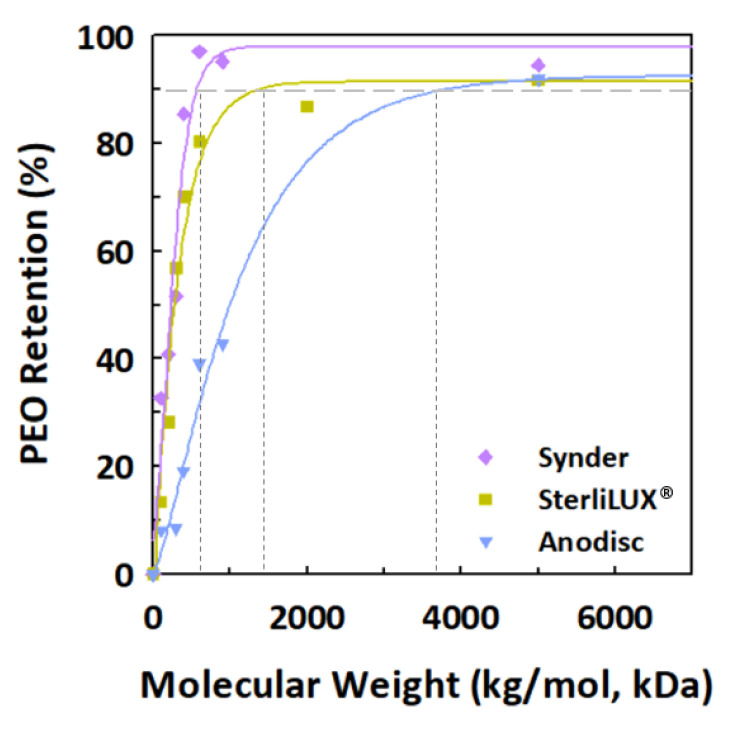
Cumulative distribution function for polyethylene oxide (PEO) solutions with a molecular weight (MW) of 100–5000 kg/mol (kDa) with the polymeric (Synder and SteriLUX^®^) and ceramic (Anodisc) membranes, and with an average pore size of 0.1 μm. Retention performance is based on measured total organic carbon (TOC) concentrations of the solutes.

We investigated the effects of two different types of particles (silica particles and PS microplastics) with the same average size (0.1 μm) on filtration and treatment performance to further understand microplastic transport compared with spherical silica particles, used for reference. Figure 1 shows a comparison of the filtration behavior of silica particles and PS microplastics for two different polymeric membranes (Synder and SteriLUX^®^) and a ceramic membrane (Anodisc) with the same nominal membrane pore size of 0.1 μm. The normalized permeate flux decline of PS microplastics was slightly greater than that of the silica particles for three different membrane filtrations, which could be explained by the steric (size) mechanism that predominates in microfiltration (MF) membranes. The distribution of particle sizes and membrane pores were thus investigated to obtain a better understanding of filtration and treatment behavior. The particle size distribution of the silica particles and the 0.1 μm-sized PS microplastics, provided by the manufacturer, is shown in Figure S2 in Supplementary Material. The results are displayed as a cumulative frequency distribution graph, showing the different peak populations of particles obtained by measuring the intensity of light scattered by a laser beam passing through a dispersed particulate sample using laser diffraction techniques. The results have also been presented as diameter (D) values, which describe the percentage of particles that are smaller than, or equal to, the percentage cut-off. The PS microplastics exhibited the narrowest particle size distribution, ranging from 0.01–0.3 μm, while the silica particles had a broader distribution of sizes, ranging from 0.01–0.8 μm. For example, the median size value (D50; 0.063 ± 0.0005 μm) in PS microplastics was smaller than 10% of the cumulative mass of silica particles (D10; 0.079 ± 0.0002 μm), while the D90 value of silica particles was twice as large as that of the PS microplastics. The estimation of the relatively accurate membrane pore sizes for three different membranes, which had a pore size of 0.1 μm, suggested by the manufacturer, was performed with PEO solutions, and achieved a 90% retention. Figure 2 shows the cumulative log-normal distribution function. Their retention results showed a PEO retention greater than 90.1% for PEO solutions of 580.6 kg/mol (Synder), PEO solutions of 1,456.1 kg/mol (SteriLUX^®^), and PEO solutions of 3652.7 kg/mol (Anodisc). The computed nominal pore sizes for the membranes were empirically estimated to be 27.3, 45.6, and 73.7 nm according to Equation (1).

Taken together, these observations suggest that the normalized permeate flux decline in PS microplastics was slightly greater than the silica particles because the distributions and diameter values were composed of particles with a small size range, which could lead to pore blocking. Furthermore, smaller membrane pores caused less flux decline for both particles, although the differences were minimal because the particles could easily penetrate through, or accumulate on, the membranes with larger pore sizes, because of relatively lower membrane resistance. The cake filtration would be expected to be the predominant fouling mechanism, because the D90 values of both silica particles and PS microplastics are larger than the estimated average pore sizes of the three different membranes. However, the results of the best fit by the blocking filtration models shown in Equations (2)–(5) with individual R^2^ values indicated that a variety of fouling behaviors, such as complete, standard, intermediate pore blocking, and cake filtration were observed, because the D10 values of the silica particles and PS microplastics are similar to the Synder and Anodisc membrane pores, respectively (Table 2). For the PS microplastics, standard pore blocking was the dominant fouling mechanism because the relatively smaller particles could easily be deposited into the internal pore walls of the membranes, while the silica particles demonstrated various fouling mechanisms, due to their wider range of particle sizes. The main fouling mechanisms were not significantly altered regardless of the membrane pores, suggesting that these fouling mechanisms were mainly determined by particle sizes.

Effluent guidelines for these particles are mandatory for wastewater discharged from domestic wastewater treatment facilities based on the performance of treatment technologies. We tried to determine whether some of the effluent guidelines could be used as an indirect measurement by evaluating any correlations with microplastics, based on the relatively facile and rapid measurements of water quality parameters in engineered systems, such as turbidity, TS, and particle size distribution. For example, linear correlations were observed between increasing feed concentration and higher values of turbidity and TS for silica particles, and PS microplastics. The turbidity values of feed water were 29.1 ± 0.1 NTU for silica particles and 105.3 ± 0.5 NTU for PS microplastic, despite being prepared with the same concentrations in an aqueous solution at neutral pH; while TS concentrations had similar values. The changes in turbidity and TS concentrations during the three different membrane filtrations were evaluated as retention performance, resulting in removal of over 90% for all membranes (Figure S3 in Supplementary Material). The turbidity retention for silica particles increased as membrane pore size decreased, while there were no significant changes in PS microplastic retention. However, turbidity, which is a qualitative characteristic that imparted by particles obstructing the transmittance of light through a water sample, has no legal bearing on wastewater effluent from treatment plants; transport of particles can be analyzed in more detail using the results of TS concentration changes. TS measurements can be useful as an indicator of the total weight of particles in wastewater samples. As with turbidity, concentrations are closely related to particle amounts, and regular monitoring of TS can help detect trends that might indicate the quantity of microplastics in wastewater samples. Any changes in TS measured during the filtration test with the membranes indicated that TS retention for silica particles and PS microplastics increased as membrane pore size increased. However, there are limitations to quantifying microplastic amounts because their total weight is difficult to distinguish from other solids suspended in water.

**Figure 3:**
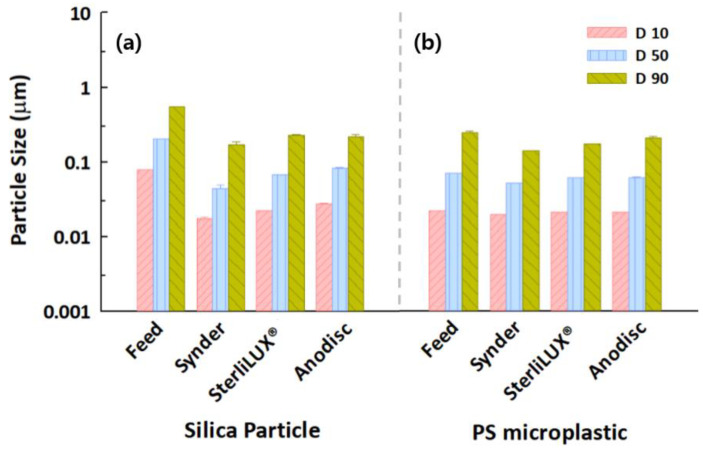
Particle size distributions in feed and permeate water samples after membrane filtration for (**a**) silica particles and (**b**) PS microplastics with an average size of 0.1 μm.

Particle size distribution analysis, which can determine and report information about the size and range of particles representative of a given material, could be one of the facile and indirect measurement indicators with which to gain insight into the transport mechanisms in membrane filtrations. This approach would not be able to directly evaluate particle removal but could evaluate specific distributions of particles both before and after membrane filtration, suggesting that the retention mechanism of particles could be determined by understanding the interactions between particle size and membrane pore. The results of the particle size distribution analysis in feed and permeate water samples that contained silica and PS microplastic with the same concentrations are shown in Figure 3. The D10 and D50 values of silica and PS microplastics for all membranes decreased significantly after membrane filtration. However, the D90 values in the permeate water samples were analyzed at 0.1700–0.2285 μm for silica particles, even though the membrane pores were estimated at 0.0273–0.0737 μm. This seems to indicate that slightly larger particles can penetrate the membrane pores due to the substantial pore size distribution of the membranes. This phenomenon was consistent with the results for the PS microplastic, meaning that size distribution analysis might be useful for understanding the behaviors of microplastics in membrane-based treatment processes.

### 3.2. Effect of Different Sizes of PS Microplastics on Filtration and Treatment Performance

**Figure 4:**
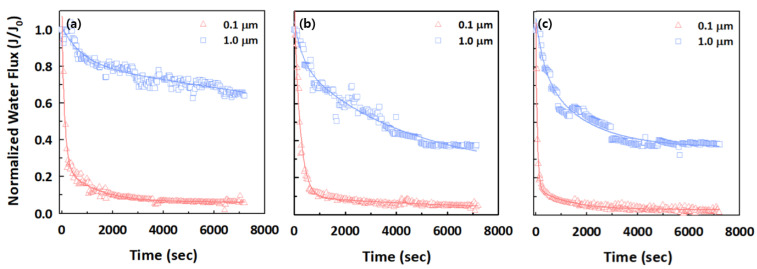
Normalized water flux decline of 0.1 and 1.0 μm PS microplastics for (**a**) Synder, (**b**) SteriLUX^®^, and (**c**) Anodisc membranes.

We investigated the effects of two different sizes (0.1 and 1.0 μm) of PS microplastics on filtration and treatment performance to understand their filtration and fouling mechanisms with polymeric and ceramic membranes. Figure 4 presents the filtration behaviors of the two different sized PS microplastics for the Synder, SteriLUX^®^, and Anodisc membranes with the same average pore size of 0.1 μm, as provided by the respective manufacturers. A relatively rapid flux decline was observed for small PS microplastics with 0.1 μm, with the normalized permeate flux suddenly decreasing after a few minutes and then consistently maintained for all membranes. These observations were attributed to standard or complete pore blocking, because the 0.1 μm PS microplastic is close to the average membrane pore size of 0.1 μm. Through blocking filtration model analysis, small PS microplastics satisfy the standard pore blocking because they can penetrate and attach to the inner wall within the membrane pores, leading to severe membrane fouling (Table 3). For the 1.0 μm PS microplastics, the normalized permeate flux at the Synder membrane, which had the smallest estimated membrane pore size, did not decrease as much as the SteriLUX^®^ and Anodisc membranes, meaning that small membrane pores might be advantageous in mitigating fouling.

**Figure 5:**
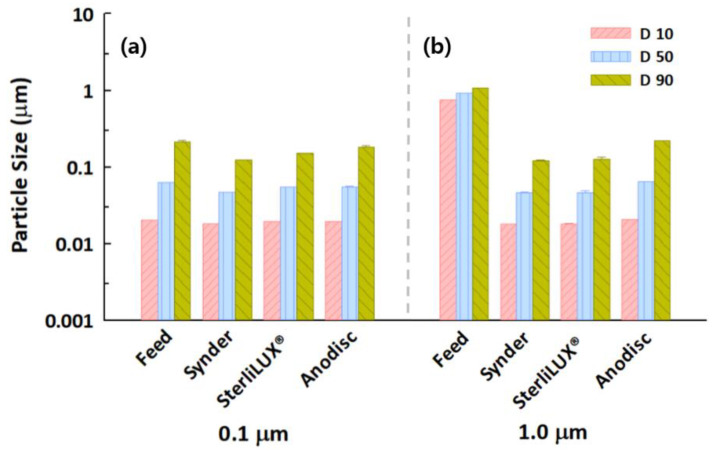
Particle size distributions in feed and permeate water samples after membrane filtration for (**a**) 0.1 μm and (**b**) 1.0 μm PS microplastics with the same concentration.

The changes in turbidity of the 0.1 and 1.0 μm PS microplastics, with the same concentration in aqueous solutions, during the three different membrane filtrations, are shown in Figure S4 in Supplementary Material. The initial turbidity values were 105.3 ± 0.5 NTU for 0.1 μm PS microplastics and 683.6 ± 1.0 NTU for 1.0 μm PS microplastics, while TS concentrations were 6.9 ± 1.9 mg/L and 4.6 ± 1.0 mg/L, respectively. As expected, the turbidity of 1.0 μm PS microplastic was almost completely removed, with over 99.6% removal by all membranes, although it showed a slightly lower retention of over 96.0% for the 0.1 μm PS microplastics. There were no significant changes in the turbidity retention of both PS microplastics depending on membrane type. However, TS retention increased slightly when the relatively larger membrane pores were within the standard deviation ranges, meaning that it would be considered an inappropriate facile, and an indirect means of measurement, to quantify microplastics and to evaluate their retention in membrane processes. The results of the particle size distribution analysis showed that all diameter values (D10, D50, and D90) of the 0.1 μm PS microplastics for the three different membranes decreased slightly after filtration, although they decreased significantly for the 1.0 μm PS microplastics because the average pore size of the three membranes was 0.1 μm (Figure 5). The Anodisc membrane, which had relatively larger membrane pores, as estimated by PEO retention, showed a relatively low retention for the 1.0 μm PS microplastics, even though the smaller size portions within the 1.0 μm PS microplastics were not completely retained and detected as D90 values in the permeate water samples.

### 3.3. Effect of Different Types of Microplastics on Filtration and Treatment Performance

**Figure 6:**
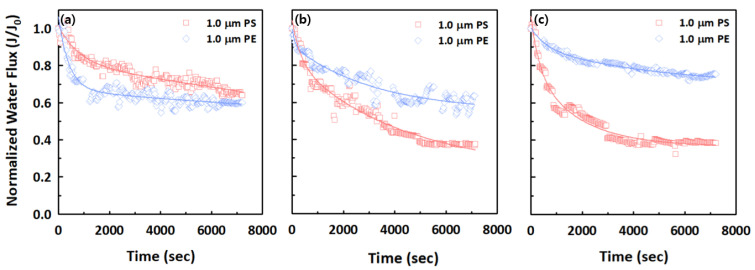
Normalized water flux decline of 1.0 μm PS and PE microplastics for (**a**) Synder, (**b**) SteriLUX^®^, and (**c**) Anodisc membranes.

The effects of two different types of 1.0 μm microplastics (PS and PE) on filtration and treatment performance were investigated to understand their filtration and fouling behaviors when using polymeric and ceramic membranes. Figure 6 shows the normalized permeate flux for the 1.0 μm PS and PE microplastics for the Synder, SteriLUX^®^, and Anodisc membranes. A relatively rapid decline in normalized permeate flux was observed for the PS microplastics at the relatively larger membrane pores (SteriLUX^®^ and Anodisc membranes), while there was only a slight decrease in normalized permeate flux at the Synder membrane. For the PE microplastics, a rapid normalized permeate flux decline was observed at Synder membrane and gradual decreases at the other two membranes. In particular, the ceramic membrane (Anodisc) did not show a severe level of membrane fouling for the PE microplastics. These observations could be described through the particle size distribution of the PS and PE microplastics, with an average size of 1.0 μm. PS microplastics exhibited the narrowest particle size distribution, at around 1.0 μm, while the particle size distribution in PE microplastics was somewhat broader, ranging from 0.08–10.0 μm (Figure S5 in Supplementary Material). As described in Section 3.2, smaller microplastics, such as the PS microplastics shown in Figure S5, could lead to significant flux reduction. However, only a gradual flux decline was observed at the Synder membrane of the relatively smaller pores, which might be attributable to a shape-dependent effect, or the properties of the polymer type, such as the residual monomer content, and may not only be caused by the size of the microplastics [ref]. The fouling mechanisms of the PS and PE microplastics for the three different membranes include complete, standard, intermediate pore blocking, and cake filtration models with R^2^ values over 0.95 for all cases, presumably due to the relatively larger microplastics (Table 4).

**Figure 7:**
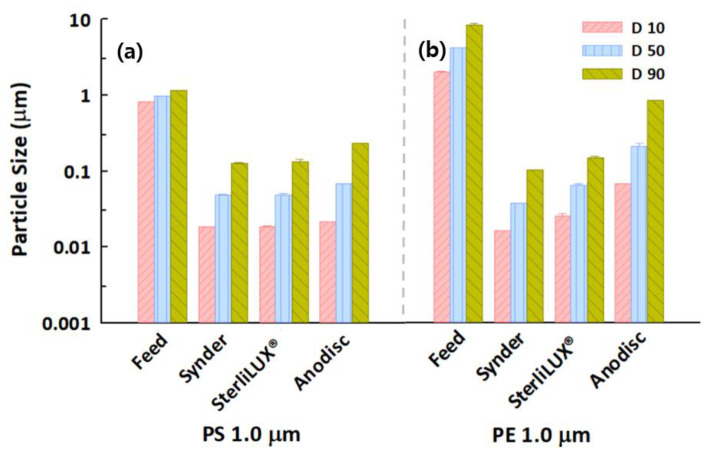
Particle size distribution in the feed and permeate water samples after membrane filtration for (**a**) 1.0 μm PS and (**b**) 1.0 μm PE microplastics with the same concentration.

The initial turbidity values were 683.6 ± 1.0 NTU for the 1.0 μm PS microplastics and 120.1 ± 3.2 NTU for the 1.0 μm PE microplastics, while TS concentrations were 4.6 ± 1.0 mg/L and 5.7 ± 0.5 mg/L, respectively. Altogether, 99% of the turbidity of 1.0 μm PS and PE microplastics was removed. However, TS retention increased slightly with increasing membrane pore size (Figure S6 in Supplementary Material). Although over 90% of TS were removed by all membranes, accurate quantification is difficult because the measurement of TS concentration has a high standard deviation range. As microplastics are extremely small particles, TS measurements showed inconsistent and inaccurate results with other facile and indirect measurement methods, as described in Section 3.1 and Section 3.2. Figure 7 shows that all diameter values of PS and PE microplastics, which were measured by particle size distribution analysis, decreased significantly after membrane filtration. However, the Anodisc ceramic membrane was relatively ineffective because the D90 value in the permeate was estimated at 0.8629 ± 0.0080 μm for the PE microplastics, even though the membrane pores were estimated at 0.0273–0.0737 μm. This was presumably caused by the differently shaped microplastics or broader particle size distribution when compared with the PS microplastics, as indicated in Figure S5.

### 3.4. Filtration Behaviors of Microplastics in Identical and Synthetic Wastewater Samples

**Figure 8:**
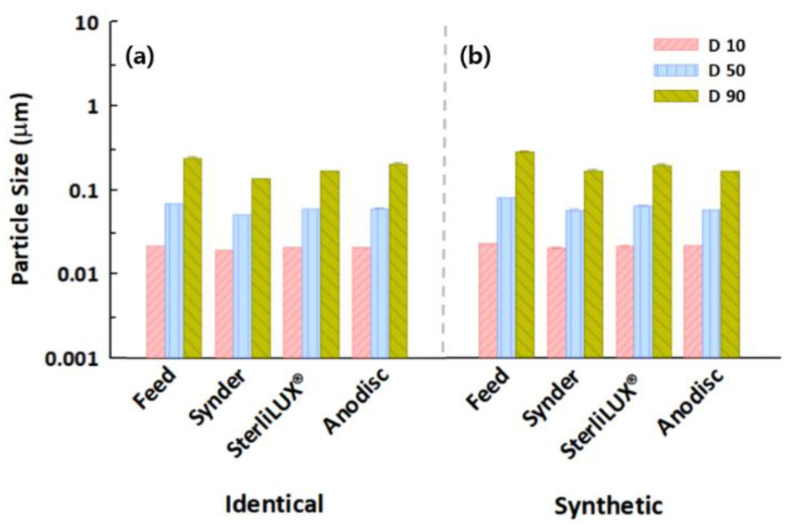
Particle size distribution in the feed and permeate water samples after membrane filtration with the diameter values of the (**a**) identical and (**b**) synthetic wastewater for the 0.1 μm PS microplastics.

**Figure 9:**
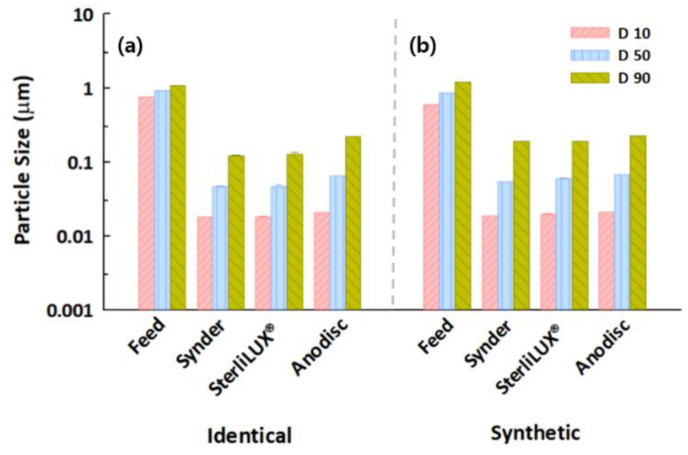
Particle size distribution in the feed and permeate water samples after membrane filtration with the diameter values of the (**a**) identical and (**b**) synthetic wastewater for the 1.0 μm PS microplastics.

**Figure 10:**
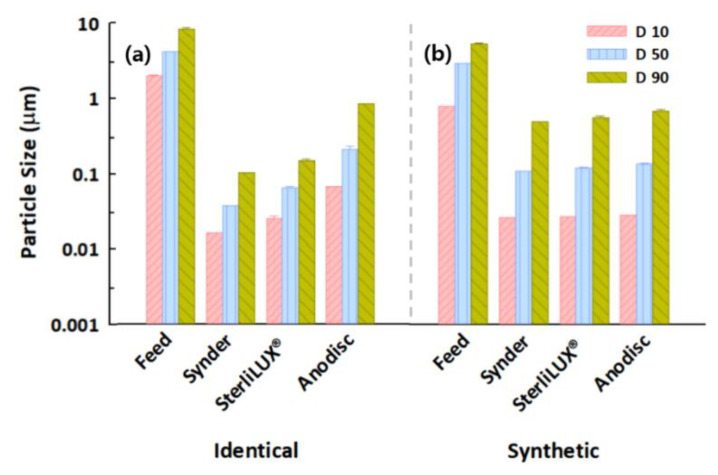
Particle size distribution in the feed and permeate water samples after membrane filtration with the diameter values of the (**a**) identical and (**b**) synthetic wastewater for the 1.0 μm PE microplastics.

A variety of physicochemical and biological compositions in wastewater samples can impact microplastic filtration behavior and retention performance in membrane filtrations. For example, dissolved organic matter in synthetic wastewater can exacerbate flux decline via membrane fouling, which increases filtration resistance because they contain relatively low concentrations of suspended particles. Our filtration and treatment experiments indicated that normalized permeate flux significantly decreased in the presence of microplastics in synthetic wastewater (Figures S7–S9). Similar normalized permeate fluxes in the presence and absence of wastewater were observed for the 0.1 μm PS microplastics because the synthetic wastewater, which is mostly composed of non-particulate dissolved organic matter, might not influence the transport of relatively small microplastics. In contrast, the results of 1.0 μm PS and PE microplastic experiments performed in synthetic wastewater revealed more rapid flux declines than in the identical PS or PE microplastic-containing suspensions. These observations might have been caused by the aggregation of relatively larger microplastics with dissolved organic matter, forming a cake layer on the membrane surface [47,48]. These results were also attributed to the morphological properties of the microplastics, which could not be accurately analyzed via particle size distribution, since they were measured as spherical particles. No significant changes were observed between the diameter values of the identical and synthetic wastewaters for the 0.1 μm PS microplastic particles, while the slightly decreased diameter values for the 1.0 μm PS and PE microplastics with the synthetic wastewaters were presumably caused by the physical or morphological properties of the relatively larger microplastics, which were affected by several constituents within the synthetic wastewater samples (Figure 8, Figure 9 and Figure 10). In addition to the effect of dissolved organic matter, a slight reduction in the diameter values in the permeate after membrane filtration was observed for the wastewater compared to the identical microplastic-containing solutions. In particular, the Anodisc membrane was effective in removing small microplastics because the D90 value in the permeate was the lowest of all the membranes, indicating that they almost completely removed the 0.1 μm PS microplastics from wastewater. However, the D90 values were not significantly reduced for the 1.0 μm PS and PE microplastics in synthetic wastewater. Regarding the comprehensive perspective for filtration and treatment performance, the Synder membrane was the most effective in removing PS microplastic, while the Anodisc was able to retain the most PE microplastics. This research may offer substantial potential for reducing membrane fouling and increasing the efficiency of microplastic retention techniques simply by using particle size distribution analysis with polymeric and ceramic membranes. However, the retention performance depends upon the physicochemical and morphological properties of the microplastics and wastewater composition. As this could limit the application of particle size distribution analysis, further research should focus on the development of tools to balance the benefits of efficient microplastic removal against the risks associated with the formation of organic complexation.

## 4. Conclusions

In this study, we used several facile water quality parameters that are widely applied in WWTPs to evaluate the filtration and treatment behaviors of microplastics. Our findings indicate that polymeric and ceramic membranes can remove a significant fraction of microplastics contained within the system by adopting the evaluation of facile water quality parameters. The reason why some microplastics remain after membrane filtration is still unknown, although it appears that a significant proportion of the microplastics remaining in the permeate can be analyzed with particle size distribution analysis. The particle size distribution analysis detailed in this study is a promising technique for quantifying the transport of tiny microplastics through wastewater treatment systems. Additionally, it can improve understanding of microplastic treatment mechanisms and give more precise estimations of the relative contribution to microplastic removal by membrane-based processes, which can subsequently assist engineers in choosing appropriate membranes for their particular objectives. Our results demonstrated that the diameter values obtained from the particle size distribution analysis could significantly promote microplastic retention between the polymeric and ceramic membranes through multiple fouling mechanisms. For example, microplastics in synthetic wastewater, which contains fewer suspended particles and more dissolved organic matter than identical microplastic-containing solutions, have a rapid flux decline curve because organic matter can more effectively limit fouling sources if they contain relatively large microplastics. The ceramic membrane was particularly effective at removing PE microplastics but was less effective in retaining PS microplastics. These facile and indirect measurement methods can be used to understand the transport of microplastics in membrane-based treatment processes as our results suggest that particle size distribution analysis could handle different sizes and types of microplastics. Currently, however, there are only limited options for directly quantifying microplastics associated with wastewaters, due to high concentrations of other suspended particles, nutrients, and organic contaminants. Additional studies involving direct quantification for a variety of microplastics are necessary to validate our findings.

## Figures and Tables

**Table 1:** Specifications of polymeric and ceramic membranes for the filtration experiments.

Type	Material	Supplier	Pore Size (μm) ^1^	Pore Size (nm) ^2^	Roughness (nm)	Pure Water Permeability (L m^−2^ h^−1^ bar^−1^)	Porosity (%)
Polymeric	PVDF	Synder	0.1	27.3	118.5	992.5	75.6
	PVDF	SteriLUX^®^	0.1	45.6	37.8	1620.9	31.6
Ceramic	Al_2_O_3_	Anodisc	0.1	73.7	28.9	2439.8	95.1

^1^ provided by supplier. ^2^ computed by Equation (1).

**Table 2:** R^2^ values corresponding to the fouling mechanisms estimated by the blocking filtration model for silica particles and PS microplastics during the three different membrane filtrations.

Particle	Membrane	Complete Pore Blocking	Standard Pore Blocking	Intermediate Pore Blocking	Cake Filtration
Silica	Synder	0.9722	0.9972	0.9884	0.9954
	SteriLUX^®^	0.9256	0.9979	0.9549	0.9733
	Anodisc	0.8303	0.9527	0.9560	0.9887
PS microplastic	Synder	0.6525	0.9825	0.7778	0.8836
	SteriLUX^®^	0.7583	0.9997	0.8946	0.8908
	Anodisc	0.7813	0.9525	0.7735	0.6617

**Table 3:** R^2^ values corresponding to the fouling mechanisms estimated by the blocking model for 0.1 and 1.0 μm PS microplastics during the three different membrane filtrations.

PS Microplastic	Membrane	Complete Pore Blocking	Standard Pore Blocking	Intermediate Pore Blocking	Cake Filtration
0.1 μm	Synder	0.6525	0.9825	0.7778	0.8836
	SteriLUX^®^	0.7583	0.9997	0.8946	0.8908
	Anodisc	0.7813	0.9525	0.7735	0.6617
1.0 μm	Synder	0.9794	1.0000	0.9793	0.9732
	SteriLUX^®^	0.9724	0.9999	0.9657	0.9565
	Anodisc	0.9603	0.9997	0.9751	0.9839

**Table 4:** R^2^ values corresponding to the fouling mechanisms estimated by the blocking model for 1.0 μm PS and PE microplastics during the three different membrane filtrations.

Microplastic	Membrane	Complete Pore Blocking	Standard Pore Blocking	Intermediate Pore Blocking	Cake Filtration
Polystylene (PS)	Synder	0.9794	1.0000	0.9793	0.9732
	SteriLUX^®^	0.9724	0.9999	0.9657	0.9565
	Anodisc	0.9603	0.9997	0.9751	0.9839
Polyethylene (PE)	Synder	0.9703	0.9997	0.9842	0.9924
	SteriLUX^®^	0.9827	1.0000	0.9813	0.9791
	Anodisc	0.9675	0.9998	0.9870	0.9874

## Data Availability

Not applicable.

## Supplementary Materials

The following supporting information can be downloaded at: https://www.mdpi.com/article/10.3390/membranes12060565/s1, Figure S1. Representative diagram of the four fouling mechanisms: (a) complete pore blocking, (b) standard pore blocking, (c) intermediate pore blocking, and (d) cake filtration. Figure S2. Volume-weighted particle size distribution and cumulative size distribution curves of (a) silica particles and (b) PS microplastics with an average size of 0.1 μm. The x-axis has a logarithmic scale. Figure S3. (a) Turbidity and (b) TS retention of silica particles and PS microplastics, with an average pore size of 0.1 μm for the Synder, SteriLUX^®^, and Anodisc membranes. Figure S4. (a) Turbidity and (b) TS retention of 0.1 μm and 1.0 μm PS microplastics with an average pore size of 0.1 μm for the Synder, SteriLUX®, and Anodisc membranes. Figure S5. Volume-weighted particle size distribution and cumulative size distribution curves of (a) PS microplastic and (b) PE microplastics with an average size of 1.0 μm. The x-axis has a logarithmic scale. Figure S6. (a) Turbidity and (b) TS retention of 1.0 μm polystyrene (PS) and polyethylene (PE) microplastics with an average pore size of 1.0 μm for the Synder, SteriLUX^®^, and Anodisc membranes. Figure S7. Normalized water flux decline of 0.1 μm PS microplastics in identical and synthetic wastewater for (a) Synder, (b) SteriLUX^®^, and (c) Anodisc membranes. Figure S8. Normalized water flux decline of 1.0 μm PS microplastics in identical and synthetic wastewater for (a) Synder, (b) SteriLUX^®^, and (c) Anodisc membranes. Figure S9. Normalized water flux decline of 1.0 μm PE microplastics in identical and synthetic wastewater for (a) Synder, (b) SteriLUX^®^, and (c) Anodisc membranes.
