# Supplementary material for: Engineered Approaches to Facile Identification of Tiny Microplastics in Polymeric and Ceramic Membrane Filtrations for Wastewater Treatment
Source: Membranes (Basel). 2022 May 28;12(6):565. doi: 10.3390/membranes12060565 (PMC9231403; doi:10.3390/membranes12060565)
Supplement: Supplementary file 1 [file membranes-12-00565-s001.zip › membranes-1737151-supplementary.pdf]

membranes

Article

Engineered Approaches to Facile Identification of Tiny Microplastics in Polymeric and Ceramic Membrane Filtrations for Wastewater Treatment

Heejin Kook and Chanhyuk Park*

Department of Environmental Science and Engineering, Ewha Womans University, Seoul 03760, South Korea
* Correspondence: chp@ewha.ac.kr (C. Park)

Citation: Kook, H.; Park, C. Engineered approaches to facile identification of tiny microplastics in polymeric and ceramic membrane filtrations for wastewater treatment. Membranes **2022**, *12*, 565. https://doi.org/10.3390/membranes12060565

Academic Editor(s): Pei Sean Goh

Received: 6 May 2022
Accepted: 27 May 2022
Published: 28 May 2022

Publisher's Note: MDPI stays neutral with regard to jurisdictional claims in published maps and institutional affiliations.

Copyright: © 2022 by the authors. Submitted for possible open access publication under the terms and conditions of the Creative Commons Attribution (CC BY) license (https://creativecommons.org/licenses/by/4.0/).

Supplementary Material

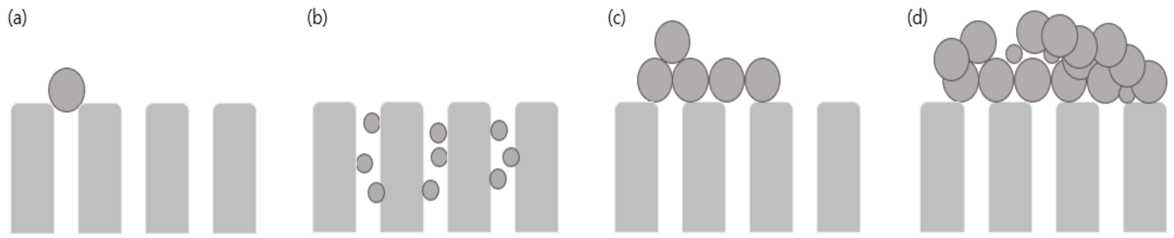

Figure S1. Representative diagram of the four fouling mechanisms: (a) complete pore blocking, (b) standard pore blocking, (c) intermediate pore blocking, and (d) cake filtration (Source: Modified from [43]).

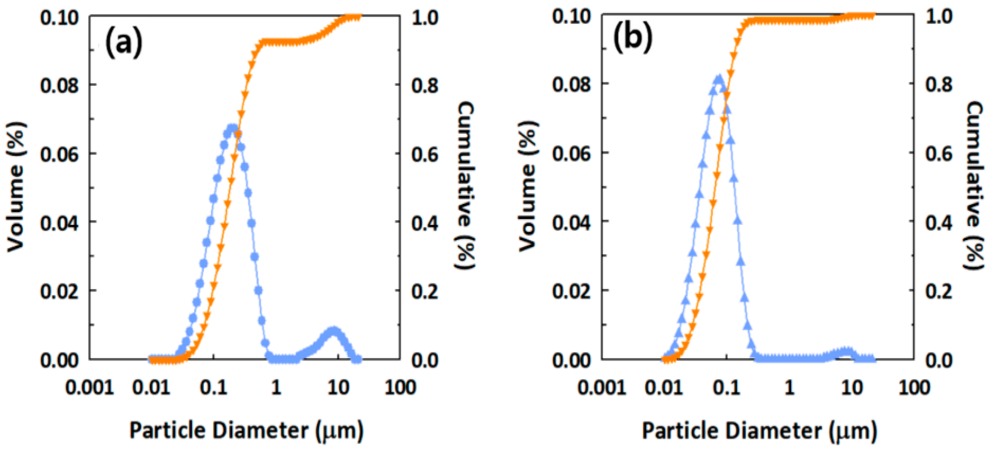

Figure S2. Volume-weighted particle size distribution and cumulative size distribution curves of (a) silica particles and (b) PS microplastics with an average size of 0.1 μm. The x-axis has a logarithmic scale.

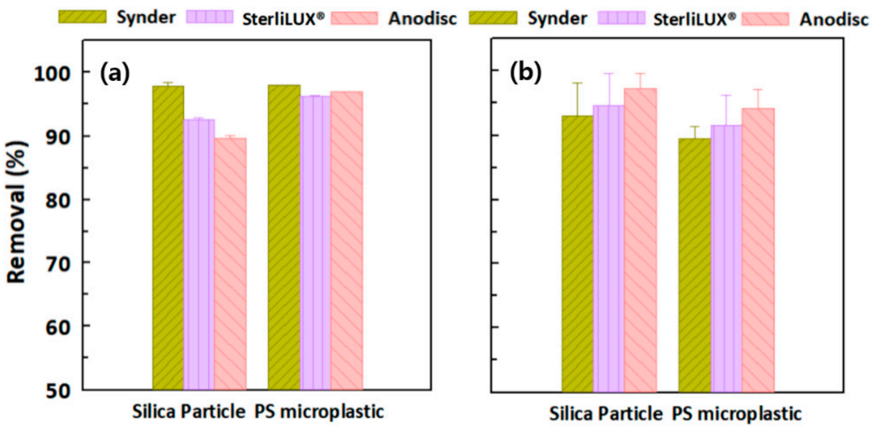

Figure S3. (a) Turbidity and (b) TS retention of silica particles and PS microplastics, with an average pore size of 0.1 μm for the Synder, SteriLUX®, and Anodisc membranes.

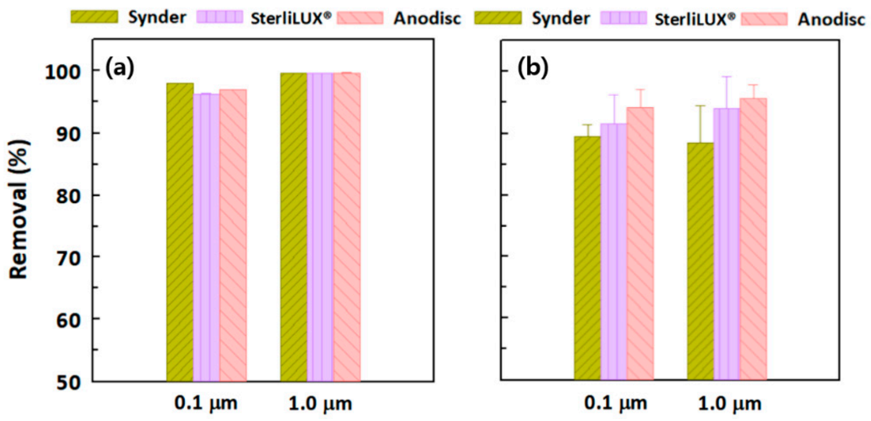

Figure S4. (a) Turbidity and (b) TS retention of 0.1 μm and 1.0 μm PS microplastics with an average pore size of 0.1 μm for the Synder, SteriLUX®, and Anodisc membranes.

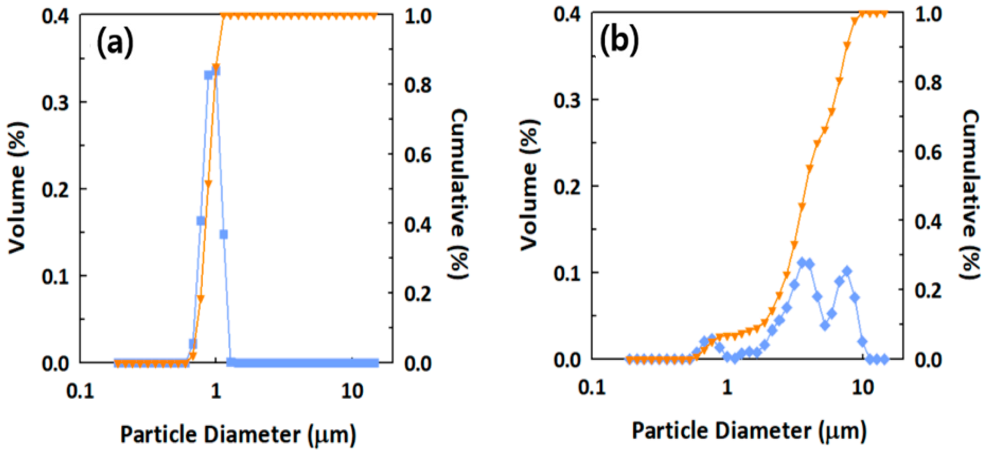

Figure S5. Volume-weighted particle size distribution and cumulative size distribution curves of (a) PS microplastic and (b) PE microplastics with an average size of 1.0 μm. The x-axis has a logarithmic scale.

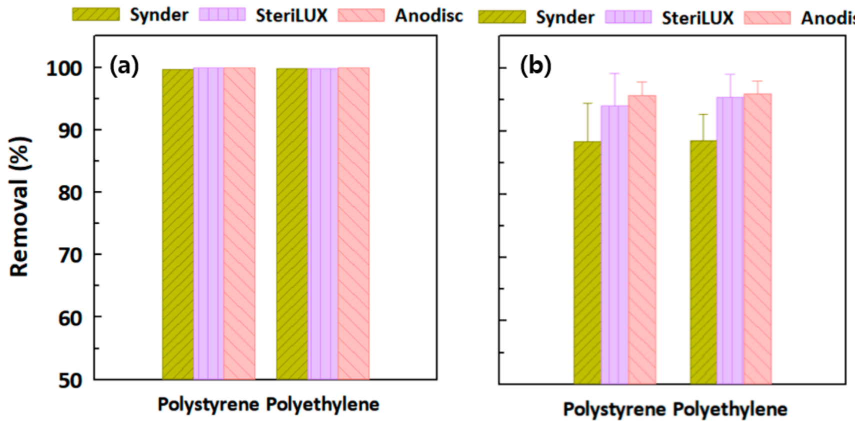

Figure S6. (a) Turbidity and (b) TS retention of 1.0 μm polystyrene (PS) and polyethylene (PE) microplastics with an average pore size of 1.0 μm for the Synder, SteriLUX®, and Anodisc membranes.

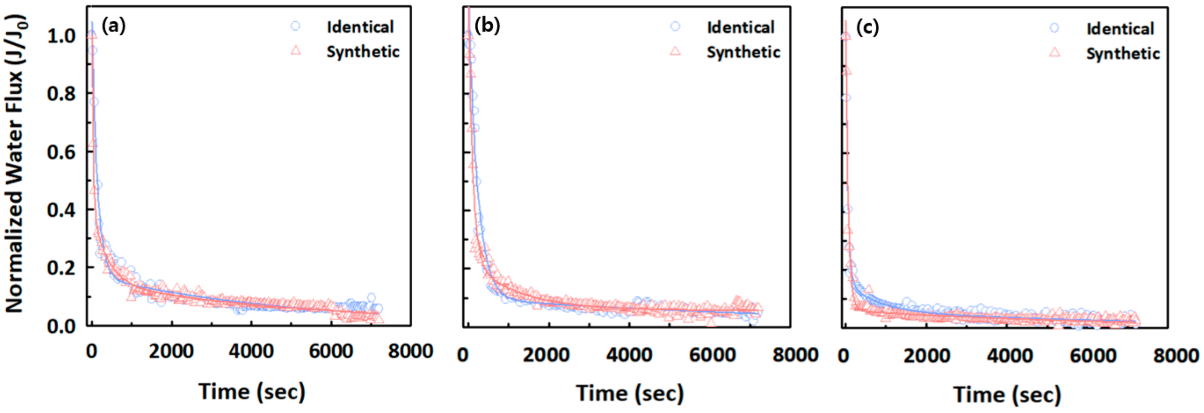

Figure S7. Normalized water flux decline of 0.1 μm PS microplastics in identical and synthetic wastewater for (a) Synder, (b) SteriLUX®, and (c) Anodisc membranes.

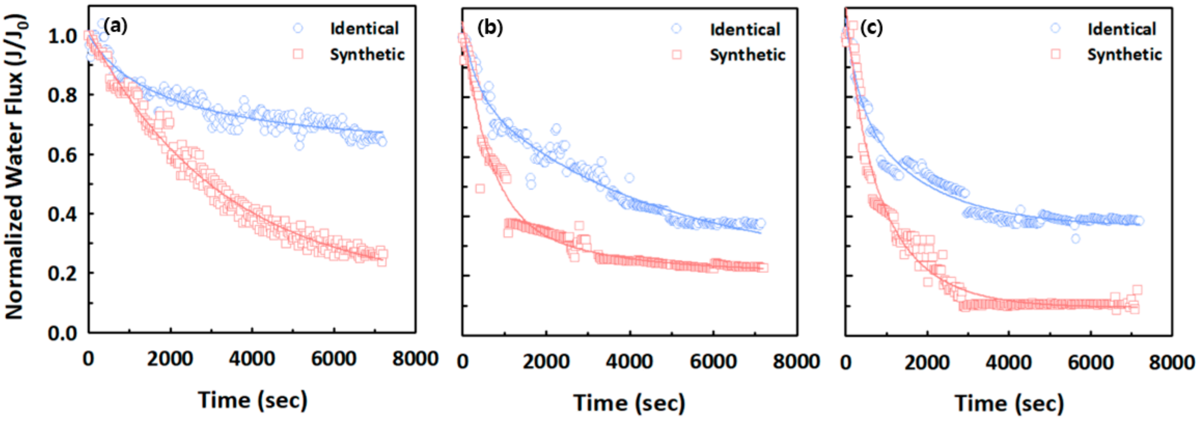

Figure S8. Normalized water flux decline of 1.0 μm PS microplastics in identical and synthetic wastewater for (a) Synder, (b) SteriLUX®, and (c) Anodisc membranes.

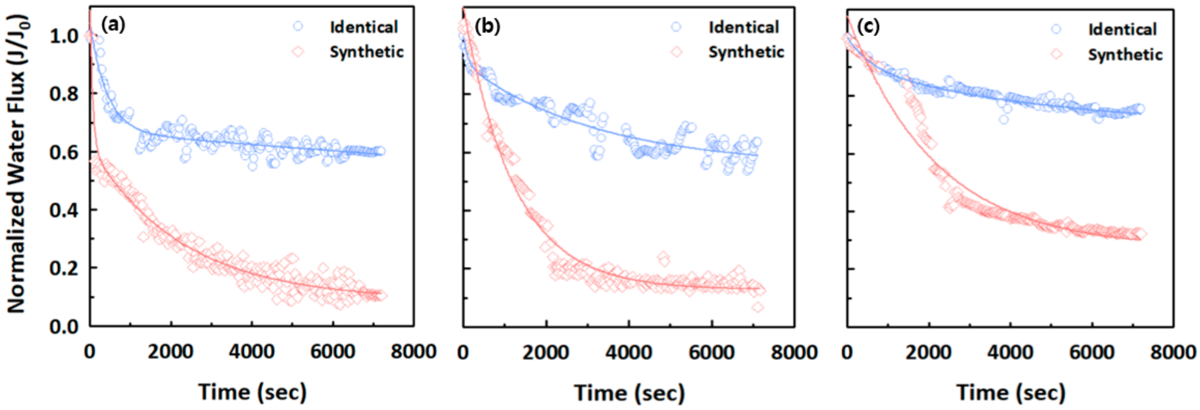

Figure S9. Normalized water flux decline of 1.0 μm PE microplastics in identical and synthetic wastewater for (a) Synder, (b) SteriLUX®, and (c) Anodisc membranes.
